# Successful desensitization to liposomal amphotericin B for the treatment of disseminated cryptococcosis in pregnancy: a case report

**DOI:** 10.1128/asmcr.00142-25

**Published:** 2025-11-12

**Authors:** Susanne O. Ajao, Julia M. Nelson, Arsheena Yassin, Emily B. Rosenfeld, Justin S. Brandt, Catherine Monteleone, Pinki J. Bhatt, Ahmed Abdul Azim

**Affiliations:** 1 Division of Allergy, Immunology, and Infectious Diseases, Department of Medicine, Rutgers Robert Wood Johnson Medical School 12287 , New Brunswick, New Jersey, USA; 2 Department of Medicine, Rutgers Robert Wood Johnson Medical School 12287 , New Brunswick, New Jersey, USA; 3 Robert Wood Johnson University Hospital 25044, New Brunswick, New Jersey, USA; 4 Division of Maternal-Fetal Medicine, Department of Obstetrics, Gynecology, and Reproductive Sciences, Rutgers Robert Wood Johnson Medical School, New Brunswick, New Jersey, USA; 5 Department of Obstetrics and Gynecology, NYU Grossman School of Medicine 12296 , New York, New York, USA; Vanderbilt University Medical Center, Nashville, Tennessee, USA

**Keywords:** cryptococcosis, HIV, AIDS, liposomal amphotericin B, amphotericin B lipid complex, allergy, desensitization, pregnancy

## Abstract

**Background:**

Cryptococcosis is the most common opportunistic fungal infection in people with human immunodeficiency virus. On a global scale, it causes 15% of acquired immunodeficiency syndrome-related mortality.

**Case Summary:**

We report the case of a 21-year-old cisgender pregnant woman living with human immunodeficiency virus who presented with headache and photophobia. Her clinical course was complicated by recurrent hospital admissions for disseminated cryptococcosis and a life-threatening reaction to liposomal amphotericin B, the mainstay of treatment. A desensitization protocol was implemented, allowing for continuation of therapy during pregnancy.

**Conclusion:**

The management of cryptococcosis in pregnancy is challenging due to the potential teratogenic effects of antifungals used and the limited therapeutic options available. This case highlights the complexities of treatment in pregnancy and demonstrates successful desensitization to liposomal amphotericin B during pregnancy.

## INTRODUCTION

Cryptococcosis, caused by the fungus *Cryptococcus neoformans* or *Cryptococcus gattii*, is an opportunistic infection associated with high mortality and morbidity in patients with advanced human immunodeficiency virus (HIV) (1, 2). It is estimated that there are 152,000 cases and 112,000 deaths annually due to cryptococcal meningitis in people with HIV (PWH) worldwide (3). Pregnancy presents a unique challenge in the treatment of cryptococcosis, as pregnant people are typically excluded from research due to concerns for antifungal-associated teratogenicity. Current knowledge of cryptococcosis treatment in this patient population is limited to a few published cases and expert opinions. The patient presented in this case experienced a complicated course of disseminated cryptococcosis during her pregnancy, with a severe reaction to liposomal amphotericin B (L-AmB) requiring amphotericin desensitization.

## CASE PRESENTATION

A 21-year-old G2P1 cisgender woman at 16 weeks and 5 days of gestation with a history of uncontrolled congenital HIV and recently diagnosed disseminated cryptococcosis (diagnosed at 9 weeks of gestation) presented to our institution with left-sided headache and photophobia concerning for relapsed cryptococcal disease. Laboratory evaluation revealed a CD4^+^ count of 9 cells/mm^3^ and an HIV-1 RNA viral load of 31,600 copies/mL. Several weeks prior, she was started on single-agent L-AmB 4 mg/kg once daily induction therapy; however, this was terminated prematurely due to worsening renal function and gastrointestinal intolerance. At 13 weeks and 5 days of gestation, she began treatment with oral fluconazole 400 mg daily and antiretroviral therapy (ART) with bictegravir, tenofovir alafenamide, and emtricitabine, which had initially been delayed due to concerns for immune reconstitution inflammatory syndrome (IRIS).

**Fig 1:**
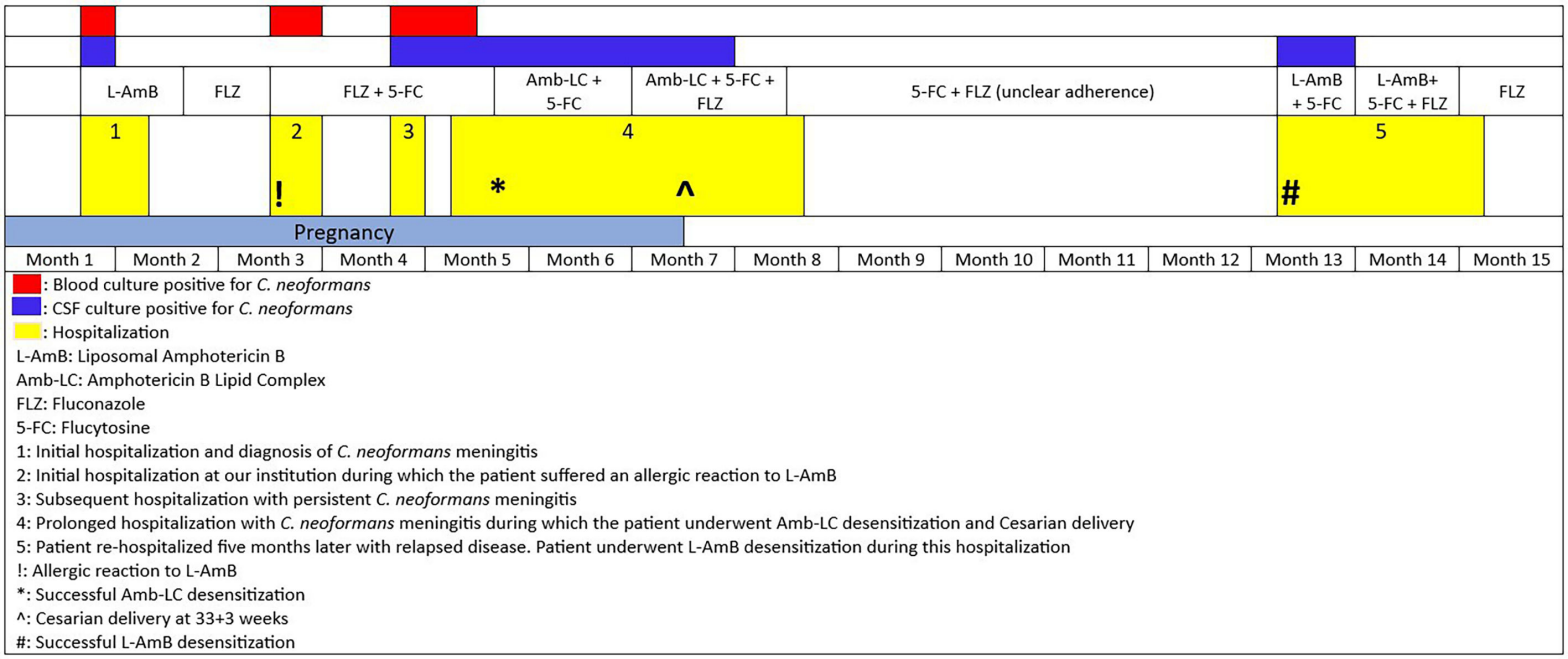
Timeline of the patient’s recurrent hospitalizations for cryptococcosis.

Although she verbalized compliance with fluconazole and ART, she was experiencing a lot of nausea and vomiting in the setting of pregnancy, raising concerns for suboptimal absorption. L-AmB and intravenous fluconazole 800 mg daily were started after admission. Within minutes of starting L-AmB, the patient became tachycardic, tachypneic, and hypoxemic. L-AmB was discontinued, and she received antihistamines and supplemental oxygen with rapid symptom improvement. L-AmB was held indefinitely, and she continued fluconazole. After discussion with the Maternal-Fetal Medicine team, flucytosine 25 mg/kg every 6 h was added as the benefits of treatment were deemed to outweigh the risks of teratogenicity in the second trimester. Blood cultures returned positive for *C. neoformans* (Fig. 1).

We consulted Allergy/Immunology regarding desensitization to L-AmB or an alternative amphotericin formulation, but given the severity of her prior reaction, desensitization to any formulation was deemed too risky. The decision was made to continue flucytosine and fluconazole. She improved, and after approximately 3 weeks of hospitalization, she left against medical advice. It is unclear if she continued taking her medications as an outpatient.

Over the next few weeks, she was readmitted multiple times for persistent symptoms, with workup revealing a low CD4^+^ count and increased HIV viral load, suggesting suboptimal medication adherence. Her blood and cerebral spinal fluid (CSF) cultures remained positive for *C. neoformans* (Fig. 1, Table 1). As her condition progressed, concerns arose about inadequate flucytosine levels due to persistent nausea and vomiting, prompting us to proceed with amphotericin B lipid complex (Amb-LC) 5 mg/kg once daily desensitization (Fig. 1). The patient was admitted to the intensive care unit (ICU) for a 10-step desensitization protocol, which she tolerated (Table 2). She continued treatment with Amb-LC and flucytosine. Thirty-four days after desensitization, she developed facial swelling and erythema, which improved with diphenhydramine and cessation of the Amb-LC infusion. She tolerated subsequent doses of Amb-LC without any additional reactions.

**TABLE 1:** Initial CSF findings on admission at each hospitalization

	Hospitalization 1	Hospitalization 3	Hospitalization 4	Hospitalization 5
Opening pressure (cm H₂O)	39	≥55	55	9
Glucose (ref: 50–75 mg/dL)	46	46	26	7
Protein (ref: 14–45 mg/dL)	32	48	48	242.5
Total nucleated cells (ref: 0–5 cells/mm^3^)	5	31	47	48
Neutrophils, absolute (cells/mm^3^)		1	7	9
Lymphocytes, absolute (cells/mm^3^)		28	35	36

**TABLE 2:** Amphotericin B lipid complex desensitization protocol

Step	Solution (mg/mL)	Rate	Time (h)	Volume infused per step (mL)	Total dose (mg)
1	0.00005	Slow push	0	5	0.00025
2	0.0005	Slow push	1	5	0.0025
3	0.005	Slow push	2	5	0.025
4	0.05	Slow push	3	5	0.25
5	0.5	Slow push over 1 min	4	5	2.5
6	0.5	Slow push over 2 min	6	20	10
7	0.5	Infuse over 10 min	8	50	25
8	1	Infuse over 20 min	10	50	50
9	1	Infuse over 2 h	12	125	125
10	1	Infuse over 2 h	24	225	225

CSF cultures remained positive for *C. neoformans*, prompting fluconazole addition to her regimen (Fig. 1). She developed severe pre-eclampsia, received peripartum zidovudine, and underwent cesarean delivery at 33 weeks and 3 days. The baby required a brief stay in the neonatal intensive care unit after birth but was otherwise healthy. Her CSF cultures cleared a few weeks after her delivery (Fig. 1). Amb-LC was discontinued 2 weeks later, and she was discharged on flucytosine and fluconazole.

Approximately 5 months later, she returned to the hospital with relapsed cryptococcal meningitis. Amb-LC was unavailable at that time, so she underwent a 12-step L-AmB desensitization without incident (Tables 3 and 4). She was initially treated with L-AmB and flucytosine; however, due to persistently positive CSF cultures and the observation that the minimum inhibitory concentration (MIC) of amphotericin for the *C. neoformans* isolate increased from 1 µg/mL during her first admission to 4 µg/mL at her fourth admission, fluconazole was added to the regimen (Fig. 1, Table 5). Approximately 6 weeks into her antifungal treatment course, and nearly 1 month after starting treatment with L-AmB, flucytosine, and fluconazole, she developed a sudden change in her mental status, and an MRI brain revealed new leptomeningeal enhancement suggestive of IRIS. CSF culture at this time was negative (Fig. 1). ART was briefly held, and she was placed on a steroid taper with clinical improvement. She was discharged after approximately 2 months of hospitalization with maintenance fluconazole therapy for at least 1 year until viral suppression and a CD4^+^ count ≥100 cells/mm^3^ were achieved. Due to past issues with adherence to oral ART, we administered one dose of injectable cabotegravir-rilpivirine prior to discharge. She has continued on this treatment since discharge, and 2.5 years later, at the time of writing, maintains an undetectable viral load with a recent CD4^+^ count of 391 cells/mm^3^. Her infant remained negative for HIV at 1 year of age.

**TABLE 3:** Liposomal amphotericin B desensitization protocol

Solution	Standard volume per bag (mL)	Drug concentration (mg/mL)
1	250	0.01
2	250	0.1
3	250	1
	Target dose = 200 mg	

**TABLE 4:** Liposomal amphotericin B desensitization protocol

Step	Solution	Rate (mL/h)	Time (min)	Volume infused per step (mL)	Dose administered with this step (mg)	Cumulative dose (mg)
1	1	1	30	0.5	0.005	0.005
2	1	2.5	30	1.25	0.0125	0.0175
3	1	5	30	2.5	0.025	0.0425
4	1	10	30	5	0.05	0.0925
5	2	2.5	30	1.25	0.125	0.2175
6	2	5	30	2.5	0.25	0.4675
7	2	10	30	5	0.5	0.9675
8	2	20	30	10	1	1.9675
9	3	5	30	2.5	2.5	4.4675
10	3	10	30	5	5	9.4675
11	3	20	30	10	10	19.4675
12	3	80	135.4	180.5325	180.5325	200
	Total time (min) = 465.4			Total time (h) = 7.756666667		

**TABLE 5:** Susceptibility profile and corresponding minimum inhibitory concentrations of *C. neoformans* isolated in CSF culture

Admission	Hospital admission #1 ARUP Laboratories (µg/mL)	Hospital admission #4 Mayo Clinic Laboratories (µg/mL)
Amphotericin B	1	4
5-Flucytosine	2	64
Fluconazole	2	≤0.12

## DISCUSSION

This case highlights the challenges associated with the treatment of disseminated cryptococcal disease in a pregnant person with advanced HIV in the setting of amphotericin allergy. Pregnancy-related immune changes may increase the risk of developing cryptococcal disease (4, 5). Pregnancy causes an immunosuppressed state to allow for the development of the fetus with an increased risk for IRIS as the immune system recovers postpartum (6). IRIS is a fatal complication of cryptococcal meningitis and the result of the exaggerated recovery of the immune system following initiation of ART (7). Patients started on treatment for cryptococcal disease during pregnancy should be monitored closely for IRIS in the postpartum period, given the risk of maternal mortality (6, 8). The ECMM/ISHAM/ASM global guideline for the diagnosis and management of cryptococcosis discourages immediate or very early initiation of ART following diagnosis of cryptococcal meningitis. A 4–6-week delay following the initiation of antifungal treatment is recommended, considering symptom improvement and intracranial pressure control (9).

Amphotericin B is the preferred antifungal agent during pregnancy, and both flucytosine and fluconazole are used cautiously due to the potential teratogenicity risk (10, 11). The ECMM/ISHAM/ASM global guideline recommends using d-AMB or L-AmB in pregnancy, as evidence suggests that they are safe during pregnancy (9). Flucytosine use in combination with amphotericin B is associated with earlier sterilization of CSF and decreased mortality 2 weeks post-treatment (12). Although flucytosine has been shown to cross the human placenta, its use in the second and third trimesters has not been associated with adverse fetal outcomes (13). Fluconazole can be initiated after delivery; however, both fluconazole and flucytosine are generally avoided in the first trimester and used judiciously in the second two trimesters based on a careful risk-benefit assessment (9). Due to limited therapeutic options in pregnancy, clear guidelines for maintenance and prophylaxis strategies have not been established.

Allergic reactions to L-AmB are rarely reported and typically occur at the start of the infusion, causing hypoxia, dyspnea, hypotension, cough, tachycardia, bronchospasms, and lip swelling that resolves with drug discontinuation (14–17). There have also been reported cases of fatal acute pulmonary reactions to the various formulations of amphotericin B (18). Presentations associated with an acute pulmonary reaction include chest pain, chest tightness, coughing, and cyanosis with new pulmonary infiltrates. This reaction occurs within the first days of treatment, usually shortly after the infusion is started, resolves when the infusion is stopped, and does not reoccur when the infusion rate is subsequently slowed with premedication (18). The mechanism of this reaction is not fully understood but is thought to be due to the liposome component, not the amphotericin B, causing a non-IgE-mediated hypersensitivity reaction. Unlike IgE-mediated (type I) allergy, the response develops on first exposure to the drug and may decrease or disappear on rechallenge of the drug (19). The patient in our case did not have symptoms commonly associated with an infusion-related reaction, such as fever, chills, flushing, nausea, or rash. Rather, she exhibited symptoms consistent with an IgE-mediated (type I) hypersensitivity reaction, including tachycardia, tachypnea, and hypoxia, despite having previously tolerated amphotericin during an earlier admission. Consequently, desensitization was pursued.

Desensitization involves administering fractional aliquots of the total therapeutic dose, beginning with a minute amount of the usual dose and increasing the dose every 15–60 min until the therapeutic dose has been reached (20, 21). During desensitization, patients may experience anywhere from mild to life-threatening reactions (22). There are no established standardized desensitization protocols for patients with previously documented reactions to the various formulations of amphotericin.

Despite the concern for teratogenicity, the patient was treated with both fluconazole and flucytosine due to the limited antifungal therapy options available during pregnancy. We failed to achieve clinical and microbiological cure in our patient with fluconazole and flucytosine; therefore, we pursued Amb-LC desensitization during pregnancy. Case reports of desensitization to other medications, such as antibiotics, have been used as guides in formulating amphotericin B desensitization protocols (23). For the first desensitization, we used Amb-LC as we suspected that the reaction may have been due to the liposome in the L-AmB. The patient was successfully desensitized to Amb-LC 5 mg/kg/day using the desensitization protocol shown in Table 2, which was similar to that provided by Shadur et al. (23). In subsequent hospitalizations, she was desensitized successfully to L-AmB 4 mg/kg/day due to the unavailability of other formulations of the drug. We used a 12-step desensitization protocol as shown in Tables 3 and 4, similar to one provided by Alandijani et al. (14) and Shadur et al. (23).

There are limited safety data and no currently established guidelines for desensitization during pregnancy (24). Most of the data in pregnant patients involve skin testing and desensitization for penicillin allergies (25, 26). Approximately 20% of pregnant patients who underwent penicillin desensitization experienced an adverse reaction, with an increased risk of adverse reactions in those with a prior positive penicillin skin test result (25). A spontaneous abortion that occurred over 10 days after desensitization was the only adverse pregnancy outcome reported (25). Additional caution is necessary in the desensitization of pregnant patients, as the physiologic changes of pregnancy can make anaphylaxis more challenging to treat, leading to increased maternal mortality rates (24, 27). An anaphylactic reaction during pregnancy may cause placental hypoperfusion and fetal distress, including fetal cerebral hypoperfusion (27). Although established guidelines for desensitization during pregnancy are lacking, emerging institutional protocols recommend involving a multidisciplinary team that includes allergists, intensivists, anesthesiologists, obstetricians, neonatologists, pharmacists, and trained nurses (24). Desensitization should be conducted in a closely monitored setting, such as an ICU or emergency department (24).

As for the decision to ultimately add fluconazole to flucytosine and amphotericin post-desensitization for persistently positive CSF cultures, we should add that certain fungal pathogens do not have well-defined antifungal breakpoints. This is the case for amphotericin against *C. neoformans*, where no clear breakpoints have been established. Epidemiological cut-off values (ECV), the MIC used to identify isolates with resistance using their phenotypic MIC value, is an alternative value used to determine wild-type isolates based on their *in vitro* response to antifungals (28, 29). While ECVs have not been established, Espinel-Ingroff et al. suggest an ECV for amphotericin against *C. neoformans* in the 0.5–1 µg/mL range (30). Our patient’s *C. neoformans* MIC was well above this value, raising concerns for acquired resistance to amphotericin and prompting a change in therapy.

### Conclusion

This case underscores the challenges of managing a complicated case of disseminated cryptococcal infection during pregnancy in PWH with limited treatment options. Additionally, we highlight the successful desensitization to amphotericin B during pregnancy. In cases of an amphotericin B reaction, close monitoring during re-exposure is essential if treatment is to be continued. A desensitization protocol should be considered to ensure safe re-exposure to the intolerant drug. Given the lack of clear guidelines for the treatment of cryptococcosis in pregnancy, there is a need for additional research into treatment options to ensure favorable maternal and fetal outcomes.
